# Profile and opinion of people with disability with respect to adapted physical activity participation in Ethiopia

**DOI:** 10.4102/ajod.v9i0.657

**Published:** 2020-09-16

**Authors:** Getachew K. Basha, Hendrik J. van Heerden

**Affiliations:** 1 School of Health Science, University of KwaZulu-Natal, Durban, South Africa; 2 College of Natural and Computational Science, University of Wollega, Nekemte, Ethiopia

**Keywords:** disability, adapted physical activity, opinion, participation, Ethiopia

## Abstract

**Background:**

Physical activity provides long-term health benefits for everyone and it is considered to play an important role in the deterioration of health predictors, such as overweight and the associated increase in cardiovascular and all-cause mortality.

**Objective:**

To explore the profile and opinion of people with disability in Ethiopia, with respect to physical activity participation.

**Method:**

The study comprised a questionnaire survey among male and female participants (*N* = 334) with visual and limb impairment, aged 15–50 years, living in urban and sub-urban areas of Ethiopia. The analyses entailed descriptive frequencies and percentages, with the chi-square statistic to test for significance between subsets of data at *p* ≤ 0.05.

**Results:**

The profile showed participants were mostly male (*n* = 221, 66.2%; *p* ≤ 0.05), had completed secondary school (*n* = 204, 61.1%; *p* ≤ 0.05), were not formally employed with some being day-labourers (*n* = 92, 27.5%) and petty traders (*n* = 71, 21.3%). The majority (*p* ≤ 0.05) had limb disabilities (*n* = 190, 57%) as opposed to vision impairment. Only 10% (*n* = 34; *p* ≤ 0.0001) confirmed participation in physical activity. More than half (*n* = 175, 52.7%; *p* ≤ 0.0001) were unsure whether exercise improves health but the majority (*n* = 175, 52.4%; *p* ≤ 0.0001) did agree that participation in adapted physical activity requires better facilities.

**Conclusion:**

Ethiopian persons with disabilities are physically inactive. There is need to raise awareness on the benefits of physical activity amongst people with disabilities and for disability friendly facilities to encourage physical activity.

## Introduction

Physical activity had a vital role in the lives of ancient cultures of the Greeks, Romans and Jewish people. The Roman physician Galen (129–210 AD) is credited as being the earliest source for describing benefits of exercise by condition and intervention details in his famous work De Sanitate Tuenda (translated by Green 1951). In the Middle Ages, Moses Maimonides (Rambam), the Spanish physician, theologian and philosopher of the 12th century, who had a major impact on the Jewish and Arabic world at that time, praised exercise as a protective factor confronting illness (Rosner 2002). The World Health Organisation (WHO 2018) defines physical activity as any bodily movement produced by skeletal muscles that requires energy expenditure – including activities undertaken whilst working, playing, carrying out household chores, travelling and engaging in recreational pursuits. Given its various advantages, physical activity is now accepted universally as a human right adopted by the (United Nations Educational, Scientific and Cultural Organization [UNESCO] 1978), through their International Charter of Physical Education and Sport (UNESCO 2018). So too, the United Nations Organization (UNO) has established the (United Nations Office on Sport for Development and Peace [UNOSDP] 2011), accountable for endorsing sport as a vehicle for attaining peace and development [United Nations Office on Sport for Development and Peace (UNOSDP) 2016].

Global initiatives, such as the World Health Organization’s Health and Development through Physical Activity and Sport (WHO 2003), the International Olympic Committee adoption of the Olympic Charter on 07 July 2007 and the United Nations Organization’s 2009 Convention on the Rights of Persons with Disabilities (article 30.5), focus on enabling persons with disabilities to participate on an equal basis with others in recreational, leisure and sporting activities (UNO 2018), and have all emphasised engaging in physical activity as a community-based rehabilitation method to maintain good health. Individuals with disabilities who participate in sports have higher self-esteem, better body image and higher rates of academic success, and are more confident and more likely to graduate from high school and matriculate in college (Lakowski & Long 2011). Physical activity and sports participation for individuals with disabilities prevent health problems by reducing the risk of developing heart disease, controlling weight, building lean muscle and reducing fat (Manley 1996). It reduces the risk of developing secondary conditions that are related to a primary disability, such as fatigue, obesity, social isolation and deconditioning (Lakowski & Long 2011). In addition to the prevention of secondary conditions and the promotion of overall health and well-being, physical activity can be important in the day-to-day life of people with disabilities. The strength and stamina that is developed by participating in physical activity can help maintain a higher level of independence. Moreover, just like able-bodied individuals, persons with disabilities have the potential to improve their physical abilities (Peynot, Chantereault & Bouizid 2011).

Participation in physical activity is related to the relative importance attached to exercise and this is influenced strongly by the, often-negative, views regarding the capabilities of persons with disabilities. Medical contra-indications to physical exercises for persons with disabilities are far less common than one might visualise. Yet, despite the many known benefits of physical exercise, several people with disability are not involved in physical activity or disability sport. Novak (2017) makes reference to ‘disability divide’ in international sport, where the increasing access to technology and sport assistance in the Global North largely benefits privileged elite disability athletes, whereas resource-constrained societies with significant economic and cultural barriers in Africa face major challenges in this respect.

Ethiopia was the first truly African Sub-Saharan country to participate at the 1968 Paralympics, when it sent two male athletes to Tel Aviv to compete in table tennis and track and field (Paralympic.org); however, in the period that followed African representation at the Paralympics did not increase notably. In that respect, the Ethiopian Paralympic Federation (2017) maintains that physical activity participation by most Ethiopian people with disability is rare and remains constrained by barriers. There is a paucity of published information in this context.

As such Kentiba and Asgedom (2017) considered elements contributing to limited participation of disabled Ethiopian children (*n* = 12) in school-based extracurricular sport activities and Mojtahedi and Katsui (2018) looked at wheelchair basketball players (*n* = 31) as a case study in advocating the implementation of the ‘right to sport’ for persons with disabilities in Ethiopia. However, that work is limited in inference, scope and in terms of sample populations; thus, the need for research on a broader scale in the Ethiopian context has been evident.

### Aim

In cognisance of the above, this article reports on the profile and opinion of people with disability in Ethiopia, with respect to adapted physical activity participation.

## Method

### Study design setting

The study entailed a descriptive survey and was conducted in the regions of Oromia & the Southern Nation and Nationality People (SNNP) Ethiopia. These areas were purposively sampled from nine regions and two city administrations in the country due them reflecting the highest prevalence rates of people with disability, in comparison with the others part of the country.

According to the Federal Democratic Republic of Ethiopia (FDRE 2002) country profile on disability, the prevalence rate of people with disability in the regional states of Ethiopia was as follows: Oromia (*n* = 333 653), Amhara (*n* = 281 291), SNNP (*n* = 174 941), Tigirayi (*n* = 90 742), Addis Ababa city administration (*n* = 45 936), Somalia (*n* = 31 686), Afar (*n* = 13 546), Benishangul (*n* = 7341), Dire Dawa city administration (*n* = 4226), Harari (*n* = 2909) and Gambela (*n* = 2581).

### Participants and sampling

The participants of the study were people with a disability in Ethiopia. The inclusion criteria were those with limb and vision impairment, aged between 15 and 50 years of age, of both genders living in urban and suburban areas, and not hospitalised. The purposive sample size was calculated by ‘RAOSOFT’ software using single population proportion formula of using 95% confidence level, 0.5% degree of precision and 50% proportion of disability prevalence. The sample size was 384 which after adding 15–20 for non-response gave an overall sample size of 400 people with disability with additional inclusive and exclusive criteria from two regions (Oromia & SNNP). The report on the Implementation of the Convention on the Right of Persons with Disabilities in Ethiopia conducted by the FDRE (2012), as referred to in the 3rd Housing and Population Census (Central Statistical Agency [CSA] 2007), indicates that vision and limb impairment have the highest disability prevalence rate (approximately *n* = 225 816; 60%) compared with all types of disability in Ethiopia within the specific age range of 15–50 years (*n* = 371 625). Accordingly, the study focused on respondents (*N* = 334) with impairment of the lower and upper limbs (*n* = 190) and visual impairment (*n* = 144).

### Ethical consideration

Ethics Committee approval was sought from and granted by the Humanities and Social Sciences Ethics Committee, University of KwaZulu-Natal (HSS/0768/015D) as well as caregiver approval for participants below the age of 18 years, in addition to gatekeeper permission from the Ethiopian Paralympic Federation. The study was conducted on the basis of ensuring the principles of privacy, confidentially, anonymity, informed consent, voluntary participation and the right to withdraw from the study.

### Data collection tools

The researcher utilised a questionnaire survey with two sections. The first section comprised a self-constructed demographic section. The second section of the questionnaire explored the opinion of participants, about adapted physical activity, using a 5-point Likert scale with items drawn from the literature reviewed in general, and in particular, adapted from Jackson (2004) and Sharkey and Gaskill (2007). The questionnaire was piloted with participants (*n* = 40) (people with disability) from one district, not included in the sampling process, with the support of one of the local sports officers. The pilot study served to determine the reliability and validity of the questionnaire. Subsequently, the questionnaire was amended by eliminating ambiguities, unnecessarily repeated questions and by ensuring that the allotted time taken to complete the questionnaire (30 min) was sufficient, in particular, to accommodate responses from visually impaired participants using enumerators to complete the questionnaire on their behalf.

### Data analysis

After the data were collected, the researcher coded raw data for subsequent analysis using Statistical Packages for Social Science (version 25). For the purpose of analysis, the 5-point Likert scale question responses were merged into three categories, namely, strongly disagree, undecided and strongly agree. Frequency count and relative frequency percentage were used as a descriptive statistic and the chi-square statistic was used in the inferential analysis for sub-sets of categorical data, with *p* ≤ 0.05 to show significant differences between data sets.

## Results

To recap, this study sought to present the profile and opinion of people with disability with respect to adapted physical activity participation in Ethiopia. The results are presented accordingly, in tabular form.

### Profile of the respondents

Table 1 reflects the demographic profile of participants.

**TABLE 1:** Demographic characteristics of participants (*N* = 334).

Characteristic	Category	*n*	%	*p*
Age range (years)	15–25	201	60.2*	0.05
	26–30	103	30.8	
	31–40	19	5.7	
	41–50	11	3.3	
Gender	Female	113	33.8	0.05
	Male	221	66.2*	
Marital status	Married	79	23.7	0.05
	Single	246	73.6*	
	Divorced	9	2.7	
Education	Primary school	62	18.5	0.05
	Secondary school	204	61.1*	
	Diploma	43	12.9	
	Degree	25	7.5	
Occupation	Day labourer	92	27.5	0.05
	Scholar/student	87	26.0	
	Petty trader	71	21.3	
	Pensioner	42	12.6*	
	Civil servant	42	12.6*	

* , significant difference.

Of the total sample population, the significant (*p* ≤ 0.05) majority presented in the 15–25 years age category (*n* = 201, 60.2%), followed by 26–30 years (*n* = 103, 30.8%), with fewer in the 31–40 years (*n* = 19, 5.7%), 41–50 years age categories (*n* = 11, 3.3%). As indicated, the sample group consisted of both female (*n* = 113, 33.8%) and male (*n* = 221, 66.2%) participants. The marital status of the respondents indicated the majority were single (unmarried) (*n* = 246, 73.6%), some married (*n* = 79, 23.7%) and the minority divorced (*n* = 9, 2.7%), respectively.

A small proportion of participants (*n* = 62, 18.5%) left school after primary education (age of 12 years), but the significant majority (*n* = 204, 61.1%; *p* ≤ 0.05) of participants concluded their education after secondary school level (age of 18 years). Some participants had completed a diploma (*n* = 43, 12.9%), and a small proportion had attained a degree (*n* = 25, 7.5%). Most participants were either day labourers (unemployed pieceworkers) (*n* = 92, 27.5%), followed by students (*n* = 87, 26%) and petty (informal) traders (*n* = 71, 21.3%). The minority were on early pension having been medically boarded (*n* = 42, 12.6%) and civil servants (*n* = 42, 12.6%), respectively, with none being employed in the private sector.

### Disability types and causes amongst respondents

Table 2 reflects the disability type of the respondents and the causes.

**TABLE 2:** Type and causes of disability (*N* = 334).

Characteristic	Category	*n*	%	*p*
Disability type	Vision	144	43.0	0.05
	Limbs	190	57.0*	
	Upper limbs	129	67.9*	0.05
	Lower limbs	61	32.1	
Disability cause	Disease	71	21.2	0.05
	Accident	154	46.1*	
	Violence	28	8.3	
	Birth	81	24.4	

* , significant difference.

From the total number of participants, significantly more (*p* ≤ 0.05) had limb disabilities (*n* = 190, 57%); comprising impairments of the lower limbs (*n* = 61, 32.1%) and upper limbs (*n* = 129, 67.9%), with fewer having vision impairment (*n* = 144, 43%); comprising blindness and low-vision. As for the causality of disability, the significant majority (*p* ≤ 0.05) was because of an accident (*n* = 154, 46.1%), followed by birth (before or during) (*n* = 81, 24.4%), disease (*n* = 71, 21.2%) and violence (*n* = 28, 8.3%) sequentially.

### Practices and opinions towards physical activity participation

Table 3 indicates the responses of participants regarding physical activity participation, with the 5-point Likert scale question responses merged into three categories, namely, strongly disagree – Likert scale 1 and 2; unsure – midpoint Likert Scale 3; and agree – Likert scale 4 and 5.

**TABLE 3:** Physical activity participation practices (*N* = 334).

Item	Disagree				Unsure				Agree				Total		*N*	*p*
	Vision		Limbs		Vision		Limbs		Vision		Limbs		Vision	Limb		
	*n*	%	*n*	%	*n*	%	*n*	%	*n*	%	*n*	%	*n*	*n*		
I often participate in physical activity	32	22.2	57	30.0	97	67.4	114	60.0	15	10.4	19	10.0	144	190	334	0.0001
I am doing moderate exercise 2–3 h per week	15	10.4	28	14.7	102	70.8	121	63.7	27	18.8	41	21.6	144	190	334	0.0001

In response to their practices in physical activity participation, the significant minority (10.0%; *p* ≤ 0.0001) of participants, irrespective of impairment, responded positively in terms of frequent participation in physical activity. Similarly, the significant minority (*p* ≤ 0.0001) of participants confirmed doing exercise of a moderate frequency accumulating to 2–3 h per week.

Table 4 indicates the responses of participants regarding opinions and perceptions of physical activity participation, with the 5-point Likert scale question responses merged into three categories, namely, strongly disagree – Likert scale 1 and 2; unsure – midpoint Likert scale 3; and agree – Likert scale 4 and 5.

**TABLE 4:** Opinions and perceptions of physical activity participation (*N* = 334).

Item	Disagree				Unsure				Agree				Total		*N*	*p*
	Vision		Limbs		Vision		Limbs		Vision		Limbs		Vision	Limb		
	*n*	%	*n*	%	*n*	%	*n*	%	*n*	%	*n*	%	*n*	*n*		
I like doing physical activity	30	20.8	12	6.3	60	41.7	164	86.3	54	37.5	14	7.4	144	190	334	0.0001
I realise physical activity improves one’s health status	55	38.2	93	48.9	87	60.4	89	46.8	2	1.4	8	4.2	144	190	334	0.0001
Participation in physical activity requires better facilities	6	4.2	108	57.4	76	52.8	77	40.5	62	43.0	113	59.4	144	190	334	0.0001

Based on their limited experience in participating in physical activity (Table 3), the significant majority (*p* ≤ 0.0001) of respondents were unsure whether they like doing physical activity and were not aware whether exercise improves health. The significant majority (*p* ≤ 0.0001) were, however, of the opinion that participation in physical activity required better facilities (Table 4).

## Discussion

### Demographic profile

The purpose of this study was to characterise Ethiopian people with limb and vision disability in terms of their profile and opinion regarding physical activity participation. Their demographic profile (Table 1) showed a dominance of males and the majority fell into the relatively young 15–25 years age category. The gender and age profile reflects the good representivity of the sample, matching the national gender-specific disability demographic, which has a larger male proportional representation amongst Ethiopians between the age of 15 and 50 years with limb and vision disabilities specifically (males: *n* = 123 333, 54.6%) and disabilities overall (males: *n* = 200 802, 54%) as documented by the UNESCO (2018). Similarly, the participants matched the national age-specific disability demographic with the highest number of people in Ethiopia, with limb and vision disabilities specifically (78 261) and disabilities overall (138 618), also falling into the age group of 15–25 years (UNESCO 2018). This is in contrast to some countries in West Africa, where Miszkurka et al. (2012) noted that mobility disability was more frequent at an older age category (35–44 years old) and more common in women than men, with a respective prevalence of 23% and 17% in Burkina Faso, 23% and 12% in Mali and 34% versus 22% in Senegal, with women having higher odds of mobility difficulty than men at every age group in the three countrie*s.* The male dominance and age differences for disability in Ethiopia may reflect a different exposure profile for East African countries, in terms of occupational risk (Table 1) and road traffic accident risk (Table 2) found in our study.

Most participants were unmarried. This contrasts with the proportional married status amongst Ethiopians without disability in the age group of 15–25 years (60.8%) and for the Ethiopian population in general, including those older than 50 years (52.7%) of age (CSA 2007). The basis for the larger number of single (unmarried) participants in our study of Ethiopians with limb and vision disability is likely to be financial challenges and discrimination. Anastasiou and Kauffman (2011) have indicated that, because of socially constructed misunderstandings, members of a community in developing countries have a tendency to believe that disabled people, particularly women, cannot participate in relationships and have families. Tefera et al. (2017) reported similar negative communal attitudes in Ethiopia towards disabled women regarding relationships. However, this demographic is universal with the majority of disabled people worldwide, particularly women, being denied the likelihood of intimacy or marriage (Frohmader & Ortoleva 2013). In addition, individuals with a disability are not considered to be as productive as able-bodied persons in the community. This negative perception and marginalisation of people with disability in society makes it difficult to find a spouse, as typically, men do not marry a woman with a disability (Mohajan 2013). In line with the result of this study, Ethiopian children with disability are often excluded from mainstream educational services and, as a result, have limited opportunity to socialise with non-disabled children in the school setting (Mohajan 2013).

According to Lasonen, Kemppainen and Raheem (2005), current Ethiopian policy requires that compulsory education lasts 8 years from age 7 to 14, comprising primary school (ages 7–12 years) and the first 2 years (cycle 1) of high school (ages 13 and 14 years), which enables students to identify their interests in further education.

Thereafter, the second cycle of secondary education (ages 15–18 years) allows students to select subjects or areas of training, which will ready them sufficiently for the work place or higher education. Accordingly, Lasonen et al. (2005) indicate that of the school-aged and tertiary student population, primary school (39.9%) and secondary school (37%) enrolments make up the majority. Although the transition rate from primary to secondary education completion is good (91.4%), close to 40% of the Ethiopian population leave school after completing the last grade of primary school (Lasonen et al. 2005). In our study, almost two-thirds of the participants had completed their secondary school education as a highest qualification. However, as mirrored in the general population (Lasonen et al. 2005), only a quarter of the respondents had completed a tertiary diploma (12.9%) or degree (7.5%), although in comparison with the general population, fewer of the respondents left school prematurely after primary school (20%). It should be noted, however, as Mohajan (2013) found that Ethiopian children with disability are often excluded from mainstream education and thus their educational demographics will differ from the general population. As such, the Ethiopian National Association for the Blind runs special elementary schools, organises training activities and aims to assist persons with visual disability in furthering their education and integration into Ethiopian society.

In terms of occupational status (Table 1), whilst a quarter of the participants were scholars or students, less than two-thirds were economically active, primarily as day labourers (unemployed piece-workers) or petty traders, which is less than the national demographic of more than three-quarters of the general population in the same age-band, being economically active (CSA 2007; Lasonen et al. 2005).

This confirms the WHO and World Bank (2011) World Report on Disability, Work and Employment that in developing countries, like Ethiopia, the educational level attained and employment opportunity is not good amongst persons with disabilities and that they are more likely to be poor compared with persons without disabilities. Data from WHO and World Bank (2011) for several African countries for the years 2003–2006 show that the average respective employment rate for people with disabilities is lower versus those without disability (Zambia 42.2% vs. 56.5%, South Africa 12.4% vs. 41.1% and Malawi 42.2% vs. 46.2%).

If one considers that only 12.6% of participants in our study were formally employed in the civil service, this figure corresponds with that for the disabled in South Africa (12.4%).

According to the International Labour Organization (ILO) (2013), an investigation conducted by the World Bank found that 55% of persons with disability in Ethiopia, particularly in Oromia region, depend on friends, family and neighbours for their living, whilst others create a meagre income through self-employment. Notwithstanding the efforts of the Ethiopian National Association of the Physical Handicapped (ENAPH) to provide for basic education courses and vocational rehabilitation in the areas of tailoring, agriculture, leather work and carpentry (ILO 2004), amongst the participants day labourers and petty traders together (48.8%) form part of the informal labourer sector and can, in essence, be considered as unemployed.

By comparison, CSA (2007) figures for the general economically inactive (unemployed) population of Ethiopia (ages 15–51 years) reflect as being far less (22%) than that of respondents. More recent for Ethiopia (Trading Economics 2018) confirms the situation, showing lower unemployment figures amongst the general population, with a rate of 17.40% in 2014 and 16.80% in 2015. This problem is made worse by the absence of social grants for individuals with a disability (Desta 2018). Moreover, employment segregation is still widespread in private organisations across Ethiopia (Ethiopian Centre for Disability and Development 2017). Advocates for Human Rights (2016), a non-governmental organisation in special consultative status with Ethiopia’s Compliance with the Convention on the Rights of Persons with Disabilities (Economic and Social Council), declares that the laws against segregation founded on disability in the service sphere are ‘limited to civil service institutions’, and are non-existent in private organisations.

According to this source, the government does not ‘take any measure against private institutions’ when they exclude workers or job seekers with disabilities. According to the Advocates for Human Rights (2016), Ethiopia’s labour proclamation legislation (article 27: work and employment) does not shield Ethiopian persons with disabilities. On the contrary, the regulation permits disability as grounds for job cancellation and it does not permit for an appeal against separation and withdrawal. The regulation does not compel business owners to make realistic job accommodations for workers in posts where the nature of their disability makes it difficult to perform their tasks with (Advocates for Human Rights 2016). Thus, disabled persons are not suitably absorbed into the labour-force and according to the ILO, several people with disabilities in Ethiopia sadly ‘depend on family funding and begging for their livelihoods’ (Advocates for Human Rights 2016).

### Disability type and causation

With reference to the disability type and cause (Table 2), category results show that vision impairment was less common than limb disability amongst participants and this strongly matched the proportional disability for vision (86.654; 42.5%) and limbs (117.157; 57.5%) for Ethiopians in the age group of 15–50 years (CSA 2017) but differs slightly when considering ages above 50 years, where more vision impairment (53%) is typically found (UNESCO 2018). When considering the higher relative proportion of upper versus lower limb impairments, respondents differed from limb disability profile amongst Ethiopians in the age group of 15–50 years (CSA 2017) and beyond (UNESCO 2018), where lower limb disabilities are in the majority. This could possibly be ascribed to a lack of wheelchairs and the burden of heavy, less-modern, crutches making lower-limb disability cases less mobile and less likely to present themselves for the research.

Pertaining to causes of the disability, findings amongst participants show that the majority were afflicted during an accident, including motor vehicle collisions, followed by congenital origin. The profile of the respondents differed somewhat from the aetiology of the Ethiopian disability population between the ages of 15 and 50 years for limb and vision impairment and the general disability population, where disability was more common because of disease (58%) but less incurring their disability before or at birth (10%–14%). The disability aetiology amongst participants in this study has foundation, however, from a world report on road traffic injury prevention jointly prepared by WHO and World Bank (2004), indicating that 90% of disability-adjusted life years are lost because of crashes (Peden et al. 2004). Similarly, the Global Status Report on Road Safety (Toroyan 2009), in collaboration with WHO, found that over 90% of the world’s fatalities on the road occur in low-income and middle-income countries, which have only 48% of the world’s registered vehicles. This is in line with the study conducted in Ethiopia by Tulu, Washington and King (2013), which found that Ethiopians are more likely to make use of commercial vehicles (37.8%), mini-buses (34.5%) and buses (18.22%) to support mobility needs and all of which are typically involved in road accidents. Research conducted in Ethiopia by Mekonnen and Teshager (2014) is congruent with the finding of this study and confirmed that road traffic accidents are a major but neglected public health challenge (Mekonnen & Teshager 2014).

### Opinions and perceptions of physical activity

Involvement in sport, exercise and other forms of leisure-time physical activity (LTPA) produces several health advantages amongst people with physical disabilities. However, the mainstream of people living with a physical disability does not partake in adequate LTPA to attain health benefits (Carroll et al. 2014). It is evident and concerning that respondents’ participation in physical activity (Table 3) is very low and their understanding of physical activity and its perceived benefits (Table 4) is vague, reflecting a knowledge gap and a lack of public education around this aspect for people with disability. Neither limb-impaired or vision-impaired individuals were sure about their frequency, intensity and duration of exercise nor of the health benefits of physical activity. Whilst only 20% indicated that they like doing physical activity, only half of those (10%) confirmed regular participation in physical activity. Although just more than half (53%) agreed that improved facilities are required, clearly a lack of awareness of physical activity influenced their uninformed (< 5%) response relating to the realisation that physical activity improves health status. However, past reports, even from first world countries, found a smaller proportion of adults with a disability (37.7%) compared with adults without a disability (49.4%) met the national recommendations for physical activity (CDC 2007), whilst 25.6% of people with a disability reported being physically inactive during an average week compared with only 12.8% of those without a disability (Lui & Hui 2009). Carroll et al. (2014) reported that over the period 2009–2012, inactivity was more prevalent amongst American adults with disability (47.1%) versus those without disability (26.1%).

Rimmer and Marques (2012) pointed out in the *Lancet* that a lack of exercise is a serious public health concern for all people, but that people with disabilities are at much greater risk of the serious health problems associated with physical inactivity. Thus, there is a sense of urgency in the promotion of physical activity amongst people with disabilities worldwide and recently Rimmer (2017) as a leading disability advocate called for ‘Less talk and more Action’ regarding equity in active living for people with disabilities. In an African context, a study conducted in Kenya by Frantz et al. (2011) noticeably also showed that non-disabled children were more active than disabled learners in physical activity and sport. Although the disabled learners were interacting with non-disabled children at school, they still tended to be less active. Consequently, a low participation rate in physical activity and the high prevalence of secondary conditions amongst adults and youth with disabilities appears to be the norm. The results of this study are congruent with the research done by Knox et al. (2013), which emphasised that differences exist in knowledge of physical activity guidelines amongst marginalised disadvantaged population groups, with them being less knowledgeable about physical activity guidelines.

In an overview, Novak (2017) has emphasised that even though athletes with disabilities have joined mainstream sport at a rapid rate across the world, Sub-Saharan Africa remains on the periphery of disability sports participation. As such, according to the Ethiopian Legal Brief (2018), the revised Ethiopian sports policy of 1994 reflects no statements that addressed physical activity participation, particularly amongst people with disabilities.

Amusa, Toriola and Onyewadume (1999) have remarked that the status of the micro-economic and macro-economic stability, as well as native politics of a country, may be blamed for its non-support of a sport. When a country is in a political crisis, such as in Liberia, Rwanda, Ethiopia, Burundi, Somalia and South Sudan, state expenditure will consistently be turned to military efforts at the cost of sports in a nation and the situation will adversely affect the participation of communities in physical activity.

## Conclusion

In summation, the Sport and Development Organisation (2009) has reported that developing countries are largely absent in International Disability Sports competitions – overall, 23% of developing countries have not joined the Deaf-Olympics, Paralympic or Special Olympics World Games, with Africa being the region with the lowest participation rate. A recent highlight for disability sport in Ethiopia was one of their para-athletes winning the Bronze Medal in the 1500 m T46 (loss of a single upper-limb) race category, during the November 2019 World Para-Athletics Championships held in Dubai. All things considered this was an exceptional individual achievement because, as borne out in this study, the majority of Ethiopian persons with vision and limb disabilities are physically inactive and ignorant about the facility requirements and health benefits of participating in physical activity.

Physical activity is, however, vital in the lifespan of human populations across the social spectrum irrespective of age, gender, religion, politics and disability. Accordingly, the demographic profile of potential participants in adapted physical activity should assist stakeholders, such as the Ethiopian Paralympic and Olympic Committee, and government sectors, such as the Ethiopian Ministry of Education, Ministry of Labour and Social Affairs and the Ethiopian Sport Commission, to increase the level of awareness and participation in physical activity amongst people with disabilities. In addition, the need for improving the accessibility of the environment for this purpose is evident and research is required to assess the degree to which the built environment in Ethiopia has been adapted for physical activity and to identify other potential barriers to physical activity participation amongst individuals with a limb or vision disability.
